# Astragalus polysaccharide ameliorates steroid-induced osteonecrosis of the femoral head by regulating *miR-200b-3p*-mediated *Wnt/β-catenin* signaling pathway via inhibiting *SP1* expression: Astragalus polysaccharide regulates SONFH via SP1

**DOI:** 10.1186/s12891-023-06447-1

**Published:** 2023-05-10

**Authors:** Shenyao Zhang, Kefang Dong, Xiangjing Zeng, Fan Wang, Min Lu

**Affiliations:** 1 Orthopedics department, The second affiliated hospital of hunan university of Chinese medicine, Changsha, China; 2 Orthopedics department, The first affiliated hospital of hunan university of Chinese medicine, Changsha, China

**Keywords:** Astragalus polysaccharide, miR-200b-3p, SP1, Steroid-induced osteonecrosis of the femoral head, Wnt/β-catenin

## Abstract

**Background:**

Steroid-induced osteonecrosis of the femoral head (SONFH) is the necrosis of the femur bone caused by prolonged and massive use of corticosteroids. The present study probed into the significance of Astragalus polysaccharide (APS) in SONFH progression.

**Methods:**

SONFH cell model was constructed using murine long bone osteocyte Y4 (MLO-Y4) cells and then treated with APS. mRNA microarray analysis selected differentially expressed genes between control group and SONFH group. RT-qPCR determined SP1 and miR-200b-3p expression. Levels of SP1, β-catenin, autophagy-related proteins (LC3II/LC3I, Beclin1, p62) and apoptosis-related proteins (Bax, C-caspase3, C-caspase9, Bcl-2) were tested by Western blot. ChIP and luciferase reporter assays confirmed relationship between SP1 and miR-200b-3p. Fluorescence intensity of LC3 in cells was detected by immunofluorescence. Flow cytometry assessed cell apoptosis. Osteonecrosis tissues from SONFH mice were examined by HE and TRAP staining.

**Results:**

APS induced autophagy and suppressed apoptosis in SONFH cell model. APS inhibited SP1 expression and SP1 overexpression reversed effects of APS on SONFH cell model. Mechanistically, SP1 targeted miR-200b-3p to inhibit Wnt/β-catenin pathway. MiR-200b-3p depletion rescued the promoting effect of SP1 on SONFH cell model by activating Wnt/β-catenin pathway. HE staining showed that APS treatment reduced the empty lacunae and alleviated inflammation in trabecular bone of SONFH mice. TRAP staining revealed decreased osteoclasts number in SONFH mice after APS treatment.

**Conclusion:**

APS regulated osteocyte autophagy and apoptosis via SP1/miR-200b-3p axis and activated Wnt/β-catenin signaling, thereby alleviating SONFH, shedding new insights for therapy of SONFH.

**Supplementary Information:**

The online version contains supplementary material available at 10.1186/s12891-023-06447-1.

## Highlights

APS induced autophagy and reduced apoptosis in SONFH cell model.

APS suppressed SP1 level in SONFH cell model.

SP1 reversed the effects of APS on SONFH cell model.

SP1 targeted miR-200b-3p and inhibited Wnt/β-catenin signaling pathway.

MiR-200b-3p depletion overturned effects of overexpressed SP1 via Wnt/β-catenin.

**Supplementary Information:**

The online version contains supplementary material available at 10.1186/s12891-023-06447-1.

## Introduction

Corticosteroids are widely used to treat autoimmune diseases such as rheumatoid arthritis and systemic lupus erythematosus [1]. One of the most common and serious complications caused by steroid therapy is steroid-induced osteonecrosis of the femoral head (SONFH) [2]. Its incidence in patients receiving long-term steroid therapy ranges from 9 to 40%, mainly in young adults aged 30–50 [3]. SONFH seriously affects the function of the hip joint, causes hip disability, and severely affects the quality of patient life. Therefore, finding effective markers is particularly important for the treatment of SONFH. The pathogenesis of SONFH is associated with increased intraosseous pressure caused by increased adipocyte proliferation and adipogenesis in the bone marrow, which can slow blood flow in the femoral head and eventually lead to ischemic osteonecrosis [4]. Additionally, decreased activity and abnormal differentiation of bone marrow mesenchymal stem cells may be an important mechanism in the occurrence and development of SONFH [5]. However, the specific mechanism of osteonecrosis of the femoral head has not been fully elucidated. Astragalus polysaccharide (APS) is a major active ingredient of Astragalus membranaceus, and possesses various biological functions such as immune regulation, anti-tumor and anti-apoptosis [6]. Studies have demonstrated that APS participated in the progression of osteoporosis. For example, APS could alleviate postmenopausal osteoporosis in mice [7]. Moreover, APS could inhibit osteoporosis through modulating FoxO3a/Wnt signaling pathway in ovariectomized rats [8]. Recently, APS was reported to improve SONFH via regulating cell autophagy and apoptosis [9]. However, the molecular mechanisms for APS in regulating SONFH remain unclear.

Sp1 transcription factor (SP1) is identified as an important transcription factor for multiple genes [10]. A DNA microarray analysis indicated that SP1 was enriched in primary osteoporosis [11]. Moreover, SP1 played a key role in inflammatory mediated bone loss through regulating PPARα promoter activity [12]. Evidence have shown that SP1 functioned as an inhibitory factor of osteogenesis [13], speculating that SP1 might act as a possible pathogenic factor of SONFH. However, the specific mechanism has not been reported. We previously found a binding site of SP1 and miR-200b-3p by ALGGEN prediction, and we speculated that SP1 might regulate miR-200b-3p expression.

MicroRNAs (miRNAs) are small non-coding RNAs with a length of about 22 nucleotides, which modulate various cellular activities [14]. MiR-200b-3p has crucial functions during osteoporosis development. For instance, miR-200b-3p was upregulated in palmitic acid-induced osteoblast apoptosis [15]. Additionally, miR-200b-3p was upregulated in SONFH patients [16], suggesting that miR-200b-3p may be a non-invasive biomarker for SONFH.

The Wnt/β-catenin signaling is a fundamental pathway that regulates osteoblastic differentiation [17]. Accumulated evidence indicates that Wnt/β-catenin signaling pathway serves as a target gene of miRNAs [18]. MiR-200b-3p could inhibit colorectal cancer cell proliferation and induced apoptosis by inactivating Wnt/β-catenin signaling [19]. In SONFH patients, partially inactivated Wnt/β-catenin signaling could reduce osteogenic differentiation [20]. However, it’s not clear whether miR-200b-3p regulates Wnt/β-catenin signaling in SONFH.

In the present research, a cell model of SONFH was established to investigate whether APS is involved in SONFH progression and the underlying mechanism. This study hypothesized that APS might inhibit miR-200b-3p by reducing SP1 level, and activate Wnt/β-catenin signaling to improve SONFH, which may supply important insights into SONFH treatment.

## Materials and methods

### Cell culture

Murine long bone osteocyte-Y4 (MLO-Y4) cells were supplied by Procell (CL-0567, Procell Life Science&Technology Co.,Ltd. Wuhan, China) and incubated in α-MEM medium (Invitrogen, Carlsbad, CA, USA) with 10% FBS and 1% penicillin-streptomycin (Solarbio, Beijing, China) at 37 °C in 5% CO_2_.

### Construction of SONFH cell model

MLO-Y4 cells were treated with 10^− 5^ M dexamethasone (DEX, Sigma, St. Louis, MO, USA) for 72 h to construct SONFH cell model (SONFH group) [21]. MLO-Y4 cells without any treatment were termed the Control group. APS was provided by Jingzhu Biological Technology Co., Ltd. (Nanjing, China), The purity of APS is more than 95%.

### Cell transfection

For SP1 gene overexpression, plasmids (oe-SP1) were constructed by cloning the full length of SP1 gene coding region into pcDNA3.1 vector (GenePharma, Shanghai, China). Besides, miR-200b-3p inhibitor and the negative control were obtained from GenePharma (Shanghai, China). Cells (1 × 10^4^ cells/well) were seeded onto 24-well plates until reaching 70% confluence and transfected with the above constructs by use of Lipofectamine™ 3000 (Invitrogen) for 24 h following manufacturer’s protocol. The mRNA expression was detected utilizing RT-qPCR. Cells in the SONFH + APS group transfected with oe-SP1 or oe-NC were termed APS + oe-SP1 group and APS + oe-NC group, respectively. Cells in the APS + oe-SP1 group transfected with miR-200b-3p inhibitor or inhibitor NC were termed APS + oe-SP1 + miR-200b-3p inhibitor group and APS + oe-SP1 + inhibitor NC group, respectively.

### RNA extraction and real-time quantitative PCR

We used TRIzol reagent (Thermo Fisher Scientific, Waltham, MA, USA) to extract total RNA from cells. For mRNA, RNA was reverse transcribed to cDNA with PrimeScript™ RT Reagent kit (Invitrogen). For miRNA, cDNA was synthesized with commercial miRNA reverse transcription PCR kit (RiboBio, China). qPCR was conducted on ABI 7500 real‑time PCR system (Applied Biosystems, Foster City, CA, USA) with SYBR Premix Ex Taq kit (Takara, Kyoto, Japan). Primer sequences of each gene are: SP1 F: 5′-TGGGTACTTCAGGGATCCAG-3′, R: 5′-TGAGGCTCTTCCCTCACTGT-3′; miR-200b-3p F: 5′-GCTGCTGAATTCCATCTAATTTCCAAAAG-3′, R: 5′-TATTATGGATCCGCCCCCAGGGCAATGGG-3′. GAPDH or U6 was used as endogenous controls. The 2^−∆∆Ct^ method was used for data analysis.

### Western blot

Proteins were isolated from MLO-Y4 cells using RIPA lysis buffer (Beyotime, Shanghai, China). Protein concentrations were assessed by a BCA kit (Beyotime). Equal proteins were separated on a 10% SDS-PAGE, followed by transferring to a PVDF membrane (Millipore, Shanghai, China). After blocking in 5% skim milk for 1 h, membranes were cultured with primary antibodies at 4 °C overnight: LC3II/LC3I (#4108, 1:1000, Cell Signaling Technology, CST, Danvers, MA, USA), Beclin1 (ab210498, 1:1000, Abcam, Cambridge, MA, USA), p62 (ab56416, 1:1,000, Abcam), Bax (ab182733, 1:1000, Abcam), C-caspase3 (#9661, 1:1000, CST), C-caspase9 (#9509, 1:1000, CST), Bcl-2 (MA5-11757, 1:1,000, Invitrogen), SP1 (ab227383, 1:1000, Abcam), β-catenin (ab32572, 1:1,000, Abcam), GAPDH (ab9485, 1:1000, Abcam). Following rinsing three times in TBST, membranes were exposed to secondary antibodies (#7074, #7076, 1:1000, CST) for 1 h at room temperature. Blots were detected with an enhanced chemiluminescence (ECL) detection system (Beyotime). Quantification of protein bands was conducted using Image J (National Institutes of Health, Bethesda, MD, USA). GAPDH was taken as an internal reference.

### Cell viability assay

Cells (2 × 10^3^/well) from control group or SONFH group were seeded in a 96-well plate for 24 h. Cells in SONFH group were then treated with increasing concentrations of APS (2, 4, 8, 16 mg/ml) at 37 °C for 24 h. Next, 10µL of MTT dye (5 mg/mL, Sigma) was added to each well. After 4 h incubation, 100 µL of DMSO was added to each well and optical density was measured at 490 nm. Finally, the optimal concentration of 8 mg/ml APS was selected for subsequent experiments.

### Immunofluorescence staining

We fixed MLO-Y4 cells which had 4% concentration of paraformaldehyde for a period of 30 min. Afterwards, they were exposed to permeabilization with 0.5% Triton X-100, as well as blocked based on 1% BSA for half an hour. We incubated cells were with LC3 antibody (#4108, 1:500, CST) at 4 °C through the night. After rinsing three times with PBS, cells were exposed to incubation with FITC-labeled goat anti-rabbit IgG (ab6717, 1:1000, Abcam) at 37 °C for an hour. Next, we stained nuclei with 40,6-diamidino-2-phenylindole (DAPI) for a period of 5 min. After rinsing with PBS, images were captured with the use of a fluorescence microscope (Olympus, Tokyo, Japan) as well as explored by adopting Image‑Pro Plus 6.0 (Media Cybernetics).

### Cell apoptosis assay

Flow cytometry detected MLO-Y4 cell apoptosis with Annexin V-FITC/PI Apoptosis Assay Kit (Sangon Biotech, Shanghai, China). Following washing using PBS solution, cells were resuspended using 1 × Binding buffer as well as stained with 10 µl Annexin V-FITC together with 5 µl propidium iodide (PI) for half an hour in the dark. In order to perform the analysis on cells, FAC Scan flow cytometry (Beckman, CA, USA) was applied. Based on Flowjo software (Version 7.6.5, TreeStar, Ashland, OR, USA), the investigation of data was performed. Meanwhile, number of apoptotic cells was counted.

### mRNA microarray analysis

MLO-Y4 cells were treated with 10^− 5^ M DEX for 72 h to construct SONFH cell model. Then, total RNA extraction was performed with Trizol reagent (Invitrogen). Complementary DNA was synthesized and labeled, which was then purified and hybridized to microarrays (Arraystar, Rockville, MD). Microarray scanning data were extracted using Agilent Feature Extraction software. GeneSpring software V13.0 (Agilent) was used to analyze mRNA array data. Overlapping differentially expressed genes with at least 2.5-fold change in either direction were considered up - or downregulated.

### Luciferase reporter assay

The predicted sequence of SP1 containing miR-200b-3p binding sites (26 ~ 35 bp, 111 ~ 120 bp) and the mutated sequence in the predicted target sites (MUT1, MUT2) were synthesized and inserted into pmirGLO luciferase vector (Promega, Madison, WI, USA). Cells were seeded in 24-well plates and co-transfected with the above recombinant reporters and miR-200b-3p inhibitor or inhibitor control with Lipofectamine 2000 (Invitrogen) for 2 days. Luciferase activity was assessed by Dual Luciferase Assay System (Promega).

### Chromatin immunoprecipitation (ChIP) assay

Binding of SP1 to miR-200b-3p promoter was examined by ChIP assay with SimpleChIP Enzymatic Chromatin IP kit (CST). Transfected cells were crosslinked with 1% formaldehyde. Cross-linked chromatin was broken into 200 to 1000-bp fragments by ultrasonic. Chromatin was immunoprecipitated with SP1 (ab227383, Abcam) or IgG (#2729, CST) antibodies at 4 °C for 2 h. Immunoprecipitate complexes were collected with protein A/G-Sepharose beads (Roche, Basel, Switzerland). After washing, the enrichment was examined using PCR.

### Establishment of SONFH model

The C57BL/6 mice (8 weeks old; 18–22 g) were purchased from Beijing Laboratory Animal Center (Beijing, China). In twelve hours light and dark circle, all mice were free to drink and eat. We randomly divided them into following groups: Control group (n = 10, only saline was injected), SONFH group (n = 10, subcutaneous injection of 20 mg/kg methylprednisolone, MPSL for 6 weeks to induce osteonecrosis), SONFH + APS group (n = 10, after being injected with MPSL for 1 h, mice received intraperitoneally administration of APS at 100 mg/kg/d for 6 weeks). Six weeks after the model establishment, the mice were killed, and the femurs were collected. All the animal experiments were approved by the Ethical Committee of Experimental Animals of The second affiliated hospital of hunan university of Chinese medicine.

### Histological examination

Femurs of mice in each group were fixed in 4% paraformaldehyde, decalcified in 10% EDTA, and embedded in paraffin. Tissues were then sliced into 5-µm-thick sections. Serial sections were stained with hematoxylin and eosin (H&E) and then stained for tartrate-resistant acid phosphatase (TRAP) activity utilizing a TRAP assay kit (Sigma) to detect osteoclasts. Finally, the histopathological changes were recorded using an optical microscope (Olympus).

### Statistical analysis

Data was analyzed using SPSS Vision 19.0 (SPSS, Chicago, IL, USA) and recorded as means ± standard deviation (SD). All data are acquired from at least three independent experiments. We employed student’s t-test to compare between two groups. For the purpose of performing comparisons of multiple groups, one-way ANOVA was adopted. *P* < 0.05 suggested a statistical difference.

## Results

### APS induced autophagy and reduced apoptosis in DEX-treated MLO-Y4 cells

**Fig. 1:**
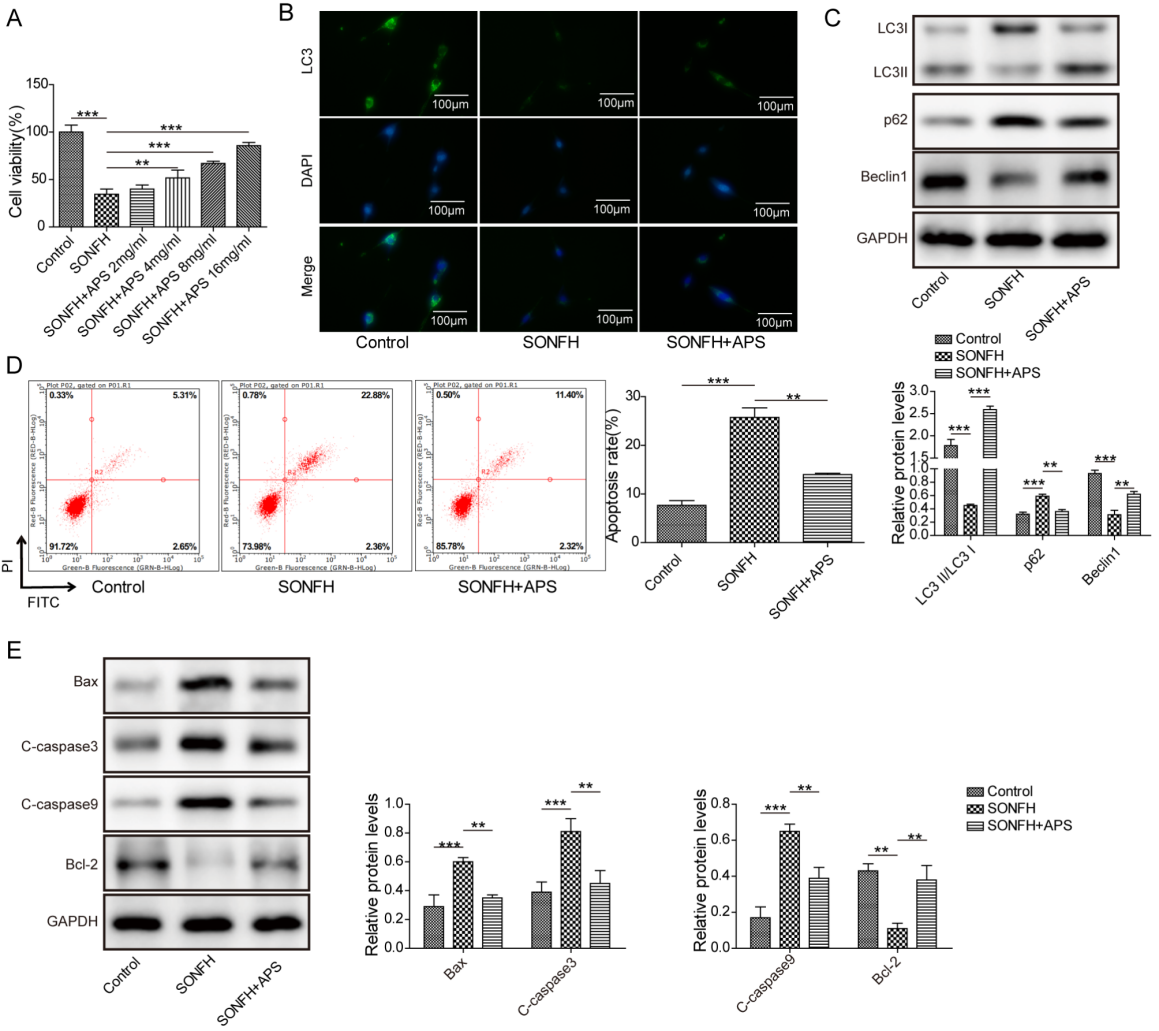
APS induced autophagy and reduced apoptosis in DEX-treated MLO-Y4 cells. MLO-Y4 cells were exposed to the treatment with 10^− 5^ M dexamethasone (DEX) for 3 days with the purpose of constructing SONFH cell model. (**A**) MTT assay examined the effect of different concentrations of APS on the viability of MLO-Y4 cells. Then, cells were subject to treatment with 8 mg/ml APS for one day. (**B**) Immunofluorescence detection of LC3 expression. (**C**) Western blot determination of autophagy-related proteins (LC3II/LC3I, Beclin1, p62). (**D**) Flow cytometry analyzed cell apoptosis. (**E**) Western blot analyzed apoptosis-related proteins (Bax, C-caspase3, C-caspase9, Bcl-2). Results expressed as mean ± SD. **P < 0.01,***P < 0.001

Firstly, MLO-Y4 cells were exposed to the treatment with 10^− 5^ M dexamethasone (DEX) for 3 days with the purpose of constructing SONFH cell model. As shown in Fig. 1A, APS treatment enhanced the viability of MLO-Y4 cells in a dose-dependent manner in SONFH cell model, and we selected an appropriate concentration of APS (8 mg/ml) for subsequent experiments. Then, cells were subject to treatment with 8 mg/ml APS for one day. Immunofluorescence analysis implied that fluorescence intensity of autophagy marker LC3 was decreased in response to DEX treatment in MLO-Y4 cells, while APS treatment abrogated this effect (Fig. 1B). Besides, Western blot results indicated that DEX treatment decreased LC3II/LC3I protein ratio and reduced Beclin1 expression, while elevated p62 level in MLO-Y4 cells. These results were reversed after APS treatment (Fig. 1C). Furthermore, cell apoptosis was obviously enhanced in SONFH group detected by flow cytometry. However, the apoptosis rate was decreased after APS treatment (Fig. 1D). Meanwhile, DEX treatment induced the expression of pro-apoptosis-related proteins Bax, C-caspase3 and C-caspase9 and reduced the level of anti-apoptosis-related protein Bcl-2, whereas these effects were abolished by APS treatment (Fig. 1E). To sum up, APS regulates autophagy and apoptosis in SONFH cell model.

### APS inhibited SP1 expression in DEX-treated MLO-Y4 cells

**Fig. 2:**
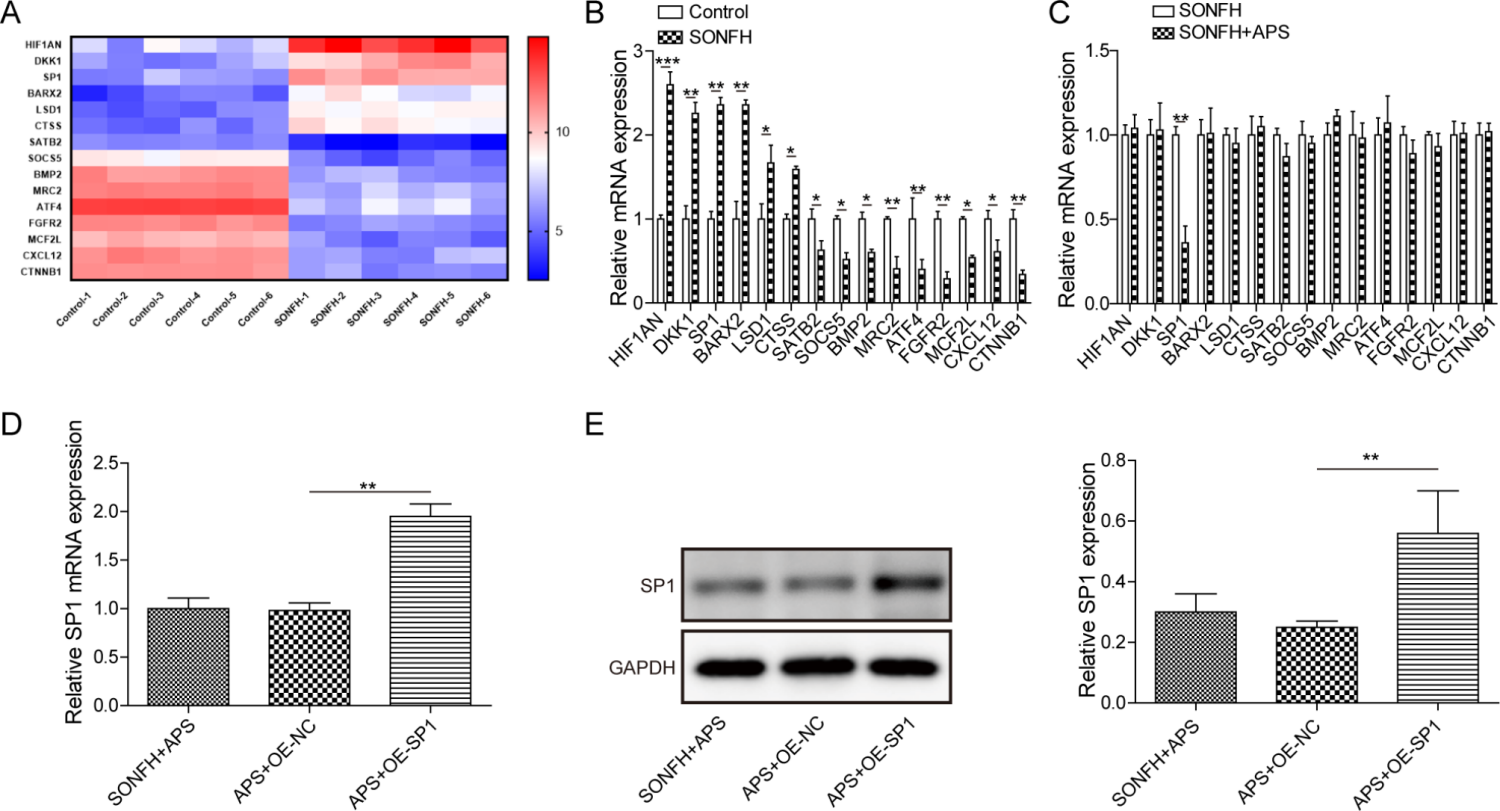
APS inhibited SP1 expression in DEX-treated MLO-Y4 cells. (**A**) mRNA microarray analysis of differentially expressed genes between control group and SONFH group. (**B**) RT-qPCR detected the content of differentially expressed genes. (**C**) RT-qPCR analyzed expression changes of each gene in SONFH cell model with/without APS treatment. (**D, E**) RT-qPCR and Western blot detection of SP1 level in APS-treated SONFH cell model with oe-SP1 (APS + oe-SP1 group) or oe-NC (APS + oe-NC group). Data are the means ± SD for three independent experiments. **P < 0.01

To further explore the underlying mechanism of APS-mediated SONFH, we analyzed the mRNA expression profiles of cells in the Control group and SONFH group. The microarray analysis identified 15 differentially expressed genes that were either upregulated or downregulated in SONFH group, compared to the Control group. Among them, 6 genes were upregulated (HIF1AN, DKK1, SP1, BARX2, LSD1, CTSS) and 9 genes were downregulated (SATB2, SOCS5, BMP2, MRC2, ATF4, FGFR2, MCF2L, CXCL12, CTNNB1) in SONFH cell model (Fig. 2A). Meanwhile, RT-qPCR tested content of differentially expressed genes and results were consistent with mRNA microarray analysis results (Fig. 2B). Additionally, RT-qPCR analysis showed that after APS treatment, SP1 mRNA was obviously decreased, but there was no significant change in the expression of other genes (Fig. 2C). Consequently, we found that SP1 overexpression elevated mRNA and protein levels of SP1 in cells with SONFH and APS treatments (Fig. 2D and E). Together, the above data indicate that APS suppresses SP1 expression in SONFH cell model.

### SP1 suppressed the effect of APS on DEX-induced MLO-Y4 cell damage

**Fig. 3:**
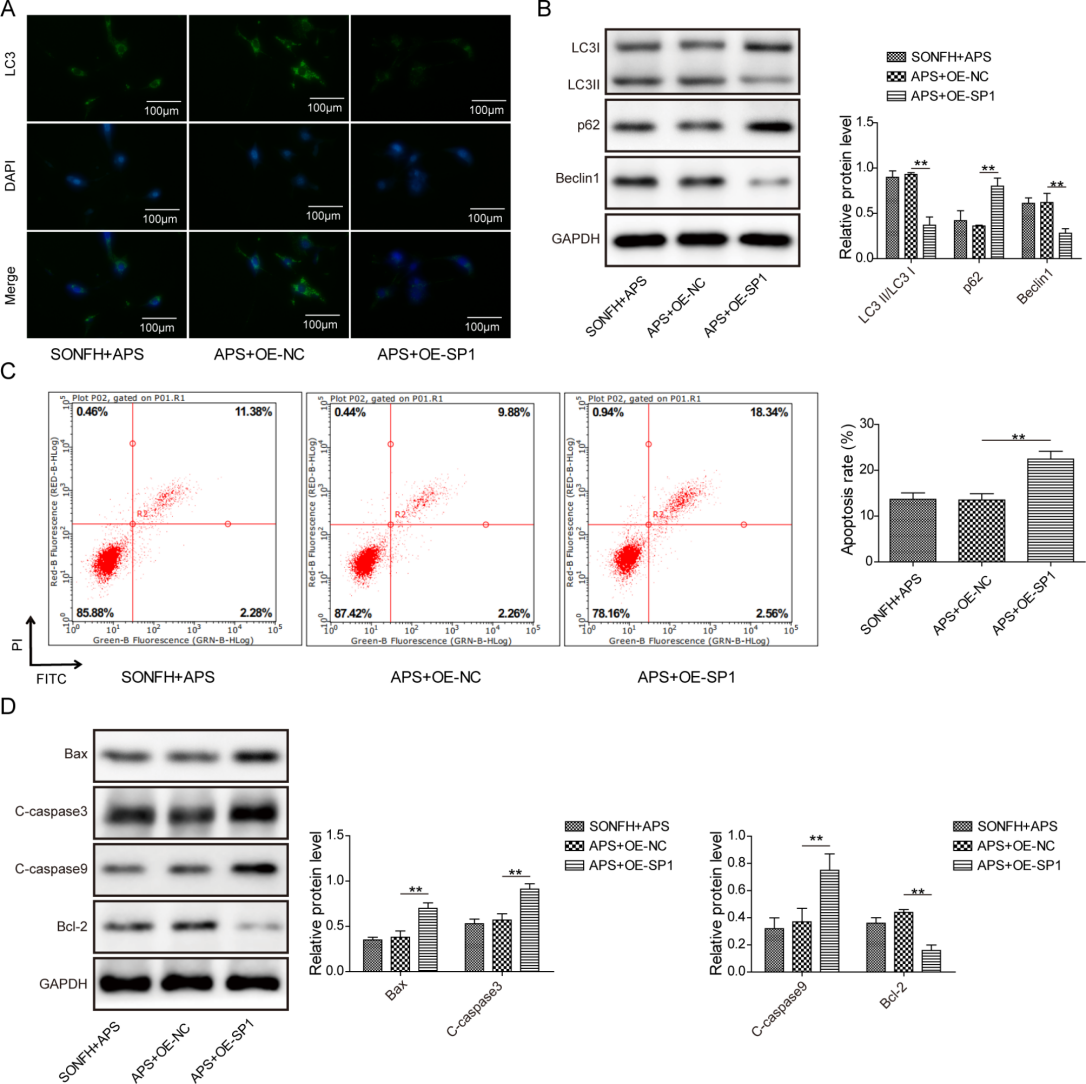
SP1 suppressed the effect of APS on DEX-induced MLO-Y4 cell damage. APS-treated SONFH cell model was transfected with oe-SP1 (APS + oe-SP1 group) or control vector (APS + oe-NC group). (**A**) Immunofluorescence detection of LC3 expression. (**B**) Western blot determination of autophagy-related proteins. (**C**) Flow cytometry analyzed cell apoptosis. (**D**) Western blot evaluated apoptosis-related proteins. Results expressed as mean ± SD. *P < 0.05, **P < 0.01

We next investigated whether APS could regulate autophagy and apoptosis in DEX-treated MLO-Y4 cells through modulating SP1. We overexpressed SP1 in APS-treated SONFH cell model. Compared to the control groups, SP1 overexpression dramatically reduced the fluorescence intensity of LC3, suggesting that autophagy was inhibited in the presence of SP1 upregulation (Fig. 3A). Additionally, overexpression of SP1 led to a significant decrease of LC3II/LC3I protein ratio and Beclin1 expression, and an increase of p62 level (Fig. 3B). On the other hand, cell apoptosis was markedly induced by SP1 upregulation (Fig. 3C). Western blot analysis further revealed that after overexpressing SP1, Bax, C-caspase3 and C-caspase9 were greatly increased, whereas Bcl-2 was decreased, implying that cell apoptosis was activated (Fig. 3D). In general, our results point out APS promotes autophagy and inhibits apoptosis in SONFH cell model through reducing SP1 level.

### SP1 inhibited Wnt/β-catenin signaling by targeting miR-200b-3p

**Fig. 4:**
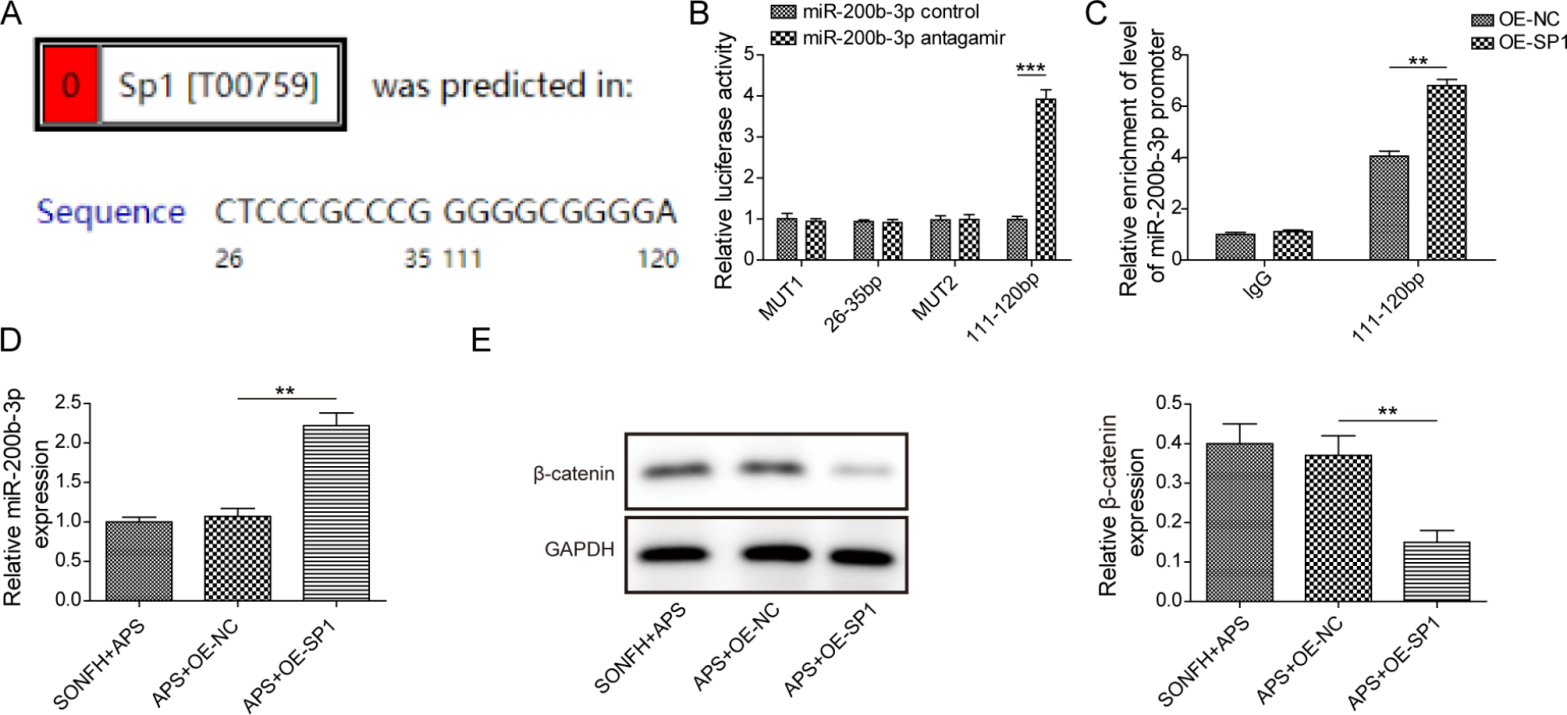
SP1 inhibited Wnt/β-catenin signaling by targeting miR-200b-3p. (**A**) ALGGEN predicted binding sequence of SP1 to miR-200b-3p promoter. (**B**) Dual luciferase reporter assay verified targeting relationship between SP1 and miR-200b-3p. (**C**) ChIP assay detected effect of SP1 on miR-200b-3p promoter. APS-treated SONFH cell model was then transfected with oe-SP1 or control vector. (**D**) RT-qPCR assessed miR-200b-3p expression. (**E**) Western blot measured β-catenin protein level. Values are mean ± SD. **P < 0.01

ALGGEN further predicted that miR-200b-3p promoter had two potential binding sites with SP1 (26 ~ 35 bp, 111 ~ 120 bp) (Fig. 4A). Luciferase reporter assay revealed that miR-200b-3p inhibitor increased the luciferase activity of 111 ~ 120 bp of SP1 significantly, while no effects were observed on the mutated forms or 26 ~ 35 bp of SP1 (Fig. 4B). Next, ChIP assay demonstrated that SP1 overexpression significantly induced the recruitment of 111 ~ 120 bp of SP1 to the miR-200b-3p promoter region **(**Fig. 4C**)**. Taken together, our results implied that the binding site was located at 111 ~ 120 bp of SP1. Subsequently, we overexpressed SP1 in APS-treated SONFH cell model, and observed that miR-200b-3p expression was obviously increased after SP1 overexpression (Fig. 4D). Importantly, SP1 upregulation significantly reduced β-catenin protein level (Fig. 4E). Overall, data above indicate that SP1 suppresses Wnt/β-catenin signaling pathway through binding to miR-200b-3p.

### Silencing miR-200b-3p weakened effect of SP1 overexpression on SONFH cell model by activating Wnt/β-catenin signaling

**Fig. 5:**
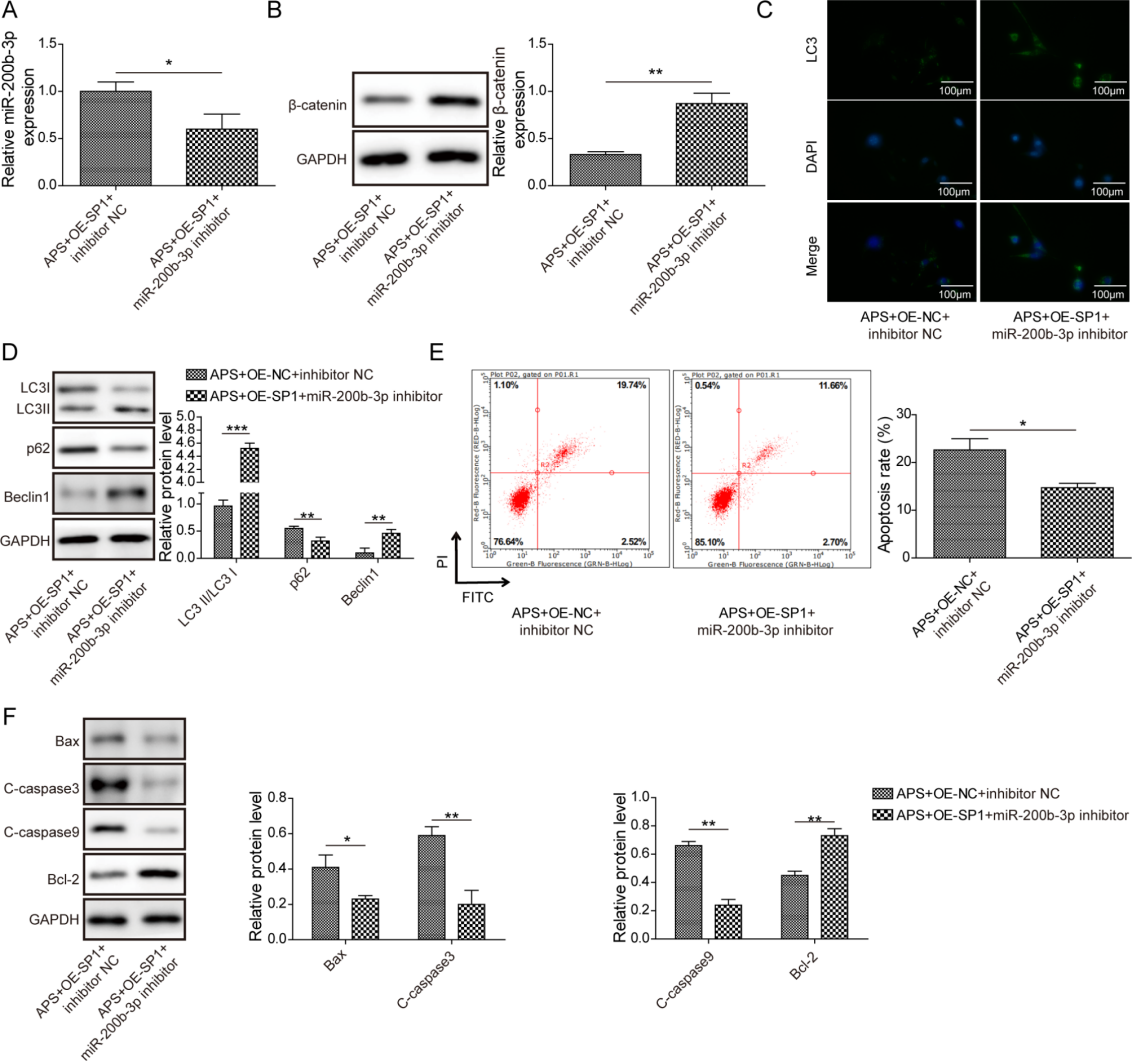
Silencing miR-200b-3p weakened effect of SP1 overexpression on SONFH cell model by activating Wnt/β-catenin signaling. APS-treated SONFH cell model was transfected with oe-SP1 and miR-200b-3p inhibitor (APS + oe-SP1 + miR-200b-3p inhibitor group) or inhibitor NC (APS + oe-SP1 + inhibitor NC group). (**A**) RT-qPCR assessed miR-200b-3p expression. (**B**) Western blot measured β-catenin protein level. (**C**) Immunofluorescence detection of LC3 expression in cells. (**D**) Western blot determination of autophagy-related proteins. (**E**) Flow cytometry analyzed cell apoptosis. (**F**) Western blot analysis of apoptosis-related proteins. Results expressed as mean ± SD. *P < 0.05, **P < 0.01

To further investigate whether SP1 regulates autophagy and apoptosis via miR-200b-3p, oe-SP1 and miR-200b-3p inhibitor were transfected into APS-treated SONFH cell model. MiR-200b-3p expression was significantly down-regulated after inhibiting miR-200b-3p (Fig. 5A). Moreover, miR-200b-3p inhibitor elevated β-catenin protein level (Fig. 5B). Furthermore, compared with the APS + oe-SP1 + inhibitor NC group, miR-200b-3p inhibition markedly increased LC3 fluorescence intensity, indicating that autophagy was activated (Fig. 5C). Meanwhile, there was an increase of LC3II/LC3I protein ratio and Beclin1expression, as well as a decrease of p62 level after suppressing miR-200b-3p (Fig. 5D). Additionally, cell apoptosis was extremely reduced after silencing miR-200b-3p (Fig. 5E). Of great importance, miR-200b-3p inhibitor reppressed Bax, C-caspase3 and C-caspase9 expression, but enhanced Bcl-2 expression, suggesting that apoptosis was suppressed (Fig. 5F). All in all, these findings reveal that miR-200b-3p inhibition suppresses the effect of SP1 upregulation on SONFH cell model through inducing Wnt/β-catenin activation.

### APS improved SONFH in mice

**Fig. 6:**
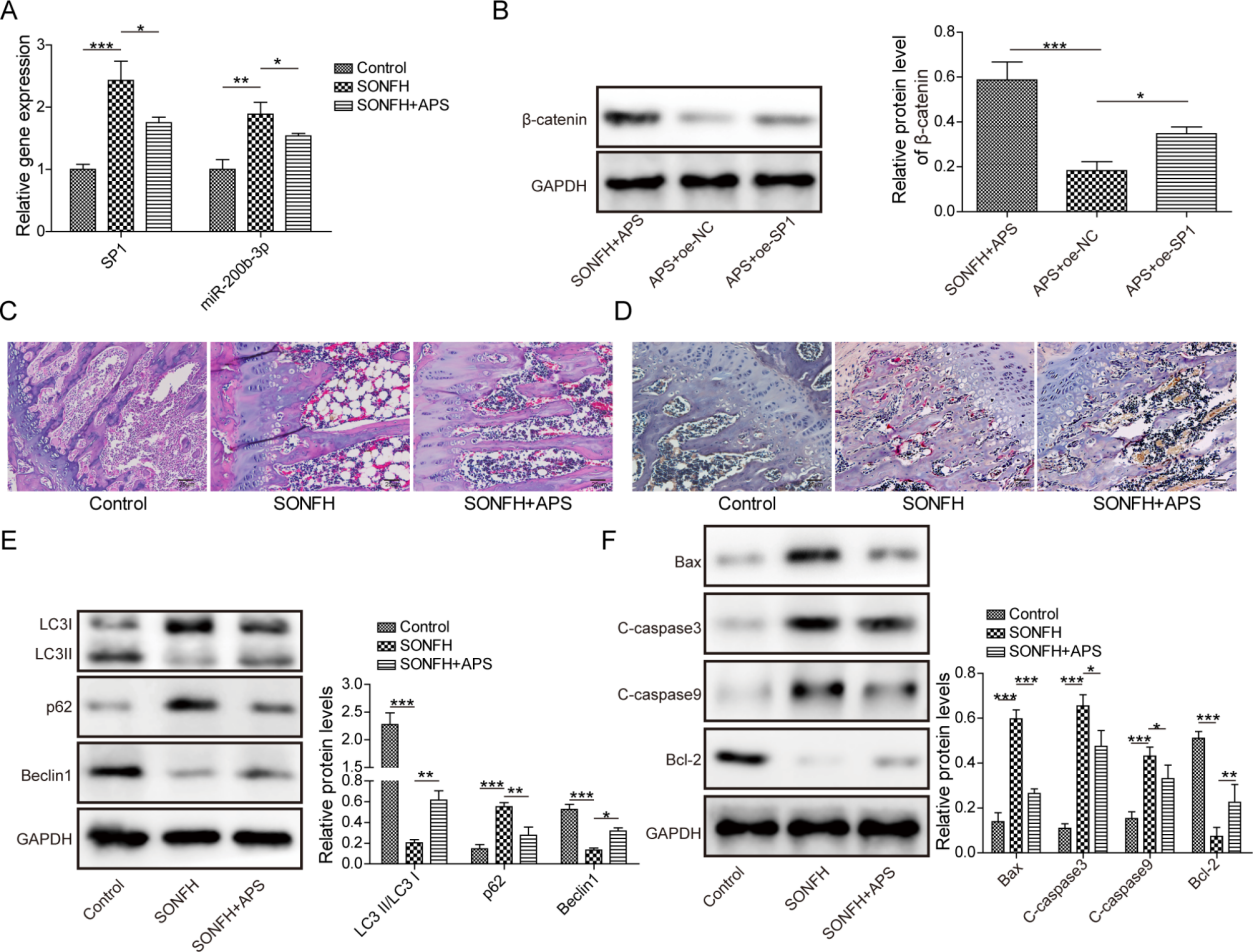
APS improved SONFH in mice. The SONFH mouse model was treated with APS (100 mg/kg/d). The mice were divided into three groups: Control group, SONFH group, SONFH + APS group. (**A**) RT-qPCR detection of SP1 and miR-200b-3p expression. (**B**) Western blot analysis of β-catenin expression. (**C**) HE staining was used to detect the pathological changes of mouse bone tissues. (**D**) TRAP staining was used to detect the number of osteoclasts in mouse bone tissues. (**E**) Western blot determination of autophagy-related proteins in osteocytes. (**F**) Western blot evaluated apoptosis-related proteins in osteocytes. Data are the means ± SD for three independent experiments. *P < 0.05,**P < 0.01,***P < 0.001

In order to explore the role of APS in SONFH progression in vivo, the SONFH mouse model was treated with APS (100 mg/kg/d). RT-qPCR and Western blot analysis showed that the increase in SP1 and miR-200b-3p expression and decrease in β-catenin expression in the bone tissues of SONFH mice were abrogated by APS treatment (Fig. 6A and B). HE staining revealed that compared with the control group, there were empty lacunae or osteocytes in the trabecular bone of SONFH mice, accompanied by inflammatory response. After APS treatment, empty lacunae was obviously reduced and inflammation was alleviated (Fig. 6C). Additionally, TRAP staining demonstrated that compared to the control mice, the number of osteoclasts in bone tissues of SONFH mice increased significantly, while decreased after APS treatment (Fig. 6D). Furthermore, autophagy was suppressed in SONFH mice, as confirmed by decreasing protein ratio of LC3II/LC3I and Beclin1 expression, while enhancing p62 protein production. These results were reversed after APS treatment (Fig. 6E). Moreover, the pro-apoptotic proteins Bax, C-caspase3 and C-caspase9 were significantly increased in SONFH mice, and the anti-apoptotic protein Bcl-2 was decreased. After APS treatment, Bax, C-caspase3 and C-caspase9 were decreased, and Bcl-2 was increased (Fig. 6F). The above results indicate that APS can alleviate SONFH in vivo.

## Discussion

So far, steroid-induced femoral head necrosis is still the most common cause of non-traumatic femoral head necrosis, with a long course and high disability rate [22]. Recently, extensive application of corticosteroids has led to an increase in the number of SONFH patients, which has brought a great burden to society [23]. Literature has revealed that steroid-induced osteocyte apoptosis caused by blood circulation disorder is a crucial pathological process in SONFH [24]. Besides, apoptosis caused by defects in autophagy is also involved in SONFH [25]. APS has the functions of enhancing immune system function, anti-inflammatory, anti-tumor, anti-oxidation and hypoglycemic [26]. Therefore, APS is essential for the prevention and treatment of diabetes and cardiovascular disease [27]. APS increased cell autophagy in vitro to suppress Parkinson [28]. Additionally, APS inhibited radiation-triggered bystander effects through suppressing apoptosis in Bone Mesenchymal Stem Cells (BMSCs) [29]. A recent research indicated that APS alleviated SONFH through promoting autophagy and inhibiting bone cell apoptosis via miR-206/HIF-1α/BNIP3 axis [9]. Our study revealed that APS could induce osteocyte autophagy and reduce osteocyte apoptosis in DEX-treated MLO-Y4 cells, suggesting that APS may alleviate SONFH progression.

SP1 is an important transcription factor and has been found to regulate osteogenic/osteoclast differentiation [30]. Notably, APS regulated the erythroid differentiation through decreasing SP1 level [31]. Here, results of mRNA microarray analysis and RT-qPCR indicated that 6 genes were increased in DEX-treated MLO-Y4 cells, and SP1 mRNA was decreased with APS treatment. Shi et al. reported that SP1 was a vital regulatory factor of hub genes in osteonecrosis of femoral head [32]. We demonstrated that SP1 reversed the effects of APS on osteocyte autophagy and apoptosis in DEX-treated MLO-Y4 cells, suggesting that APS promotes autophagy and inhibits apoptosis in SONFH cell model via reducing SP1 level.

Early studies showed that SP1 could regulate the expression of various miRNAs during bone cell differentiation, such as miR-139-5p and miR-545-3p [13, 33]. Here, by ALGGEN we predicted that miR-200b-3p promoter had two potential binding sites with SP1 (26 ~ 35 bp, 111 ~ 120 bp). Luciferase reporter assay and ChIP assay further verified that the binding site was located at 111 ~ 120 bp of SP1. Moreover, SP1 could induce miR-200b-3p expression. It has been shown that Lycium barbarum polysaccharides inhibited palmitic acid-induced osteoblast apoptosis and suppressed osteoporosis through decreasing miR-200b-3p [15]. Importantly, miR-200b-3p was increased in SONFH samples and connected with Wnt signaling pathway [16]. We next discovered that miR-200b-3p inhibitor could elevate β-catenin protein level. It has been reported that Wnt/β-catenin signalling was activated during Foxf1-mediated BMSC osteogenesis [34]. Moreover, Wnt/β-catenin inhibition suppressed osteogenic differentiation in SONFH patients [20]. Our study further demonstrated that SP1 regulated osteocyte autophagy and apoptosis in SONFH cell model through activating Wnt/β-catenin signaling through interacting miR-200b-3p, thereby improving SONFH. Previously, miR-200b-3p was reported to restrain colorectal cancer cell growth and induce apoptosis by blocking the Wnt/β-catenin pathway through targeted inhibition of Wnt1 expression [19]. Therefore, further studies are warranted to determine whether the coding gene of Wnt1 or β-catenin is the target gene of miR-200b-3p.

All together, we found APS could activate Wnt/β-catenin signaling through inhibiting SP1/miR-200b-3p axis, thereby inducing bone cell autophagy, reducing bone cell apoptosis, and improving SONFH, which may provide a new insight in function of APS as a potential therapeutic reagent for SONFH. However, lack of in vivo animal experiments and clinical data are the main limitation of the current study, which should be explored in the future.

## Electronic supplementary material

Below is the link to the electronic supplementary material.

Supplementary Material 1

## Data Availability

The datasets used and analyzed with this study are available on reasonable request from the corresponding author.

## Abbreviations

DAPI: 40,6-diamidino-2-phenylindole
APS: Astragalus polysaccharide
BMSCs: Bone Mesenchymal Stem Cells
CST: Cell Signaling Technology
ChIP: Chromatin Immunoprecipitation
DEX: Dexamethasone
HE staining: Hematoxylin and eosin staining
miRNAs: microRNAs
Y4 MLO-Y4: Murine long bone osteocyte
PI: Propidium iodide
SP1: Sp1 transcription factor
SD: Standard deviation
SONFH: Steroid-induced osteonecrosis of the femoral head
TRAP: Tartrate-resistant acid phosphatase
