# Supplementary figures and images for: Astragalus polysaccharide ameliorates steroid-induced osteonecrosis of the femoral head by regulating miR-200b-3p-mediated Wnt/β-catenin signaling pathway via inhibiting SP1 expression: Astragalus polysaccharide regulates SONFH via SP1
Source: BMC Musculoskelet Disord. 2023 May 10;24:369. doi: 10.1186/s12891-023-06447-1 (PMC10170750; doi:10.1186/s12891-023-06447-1)

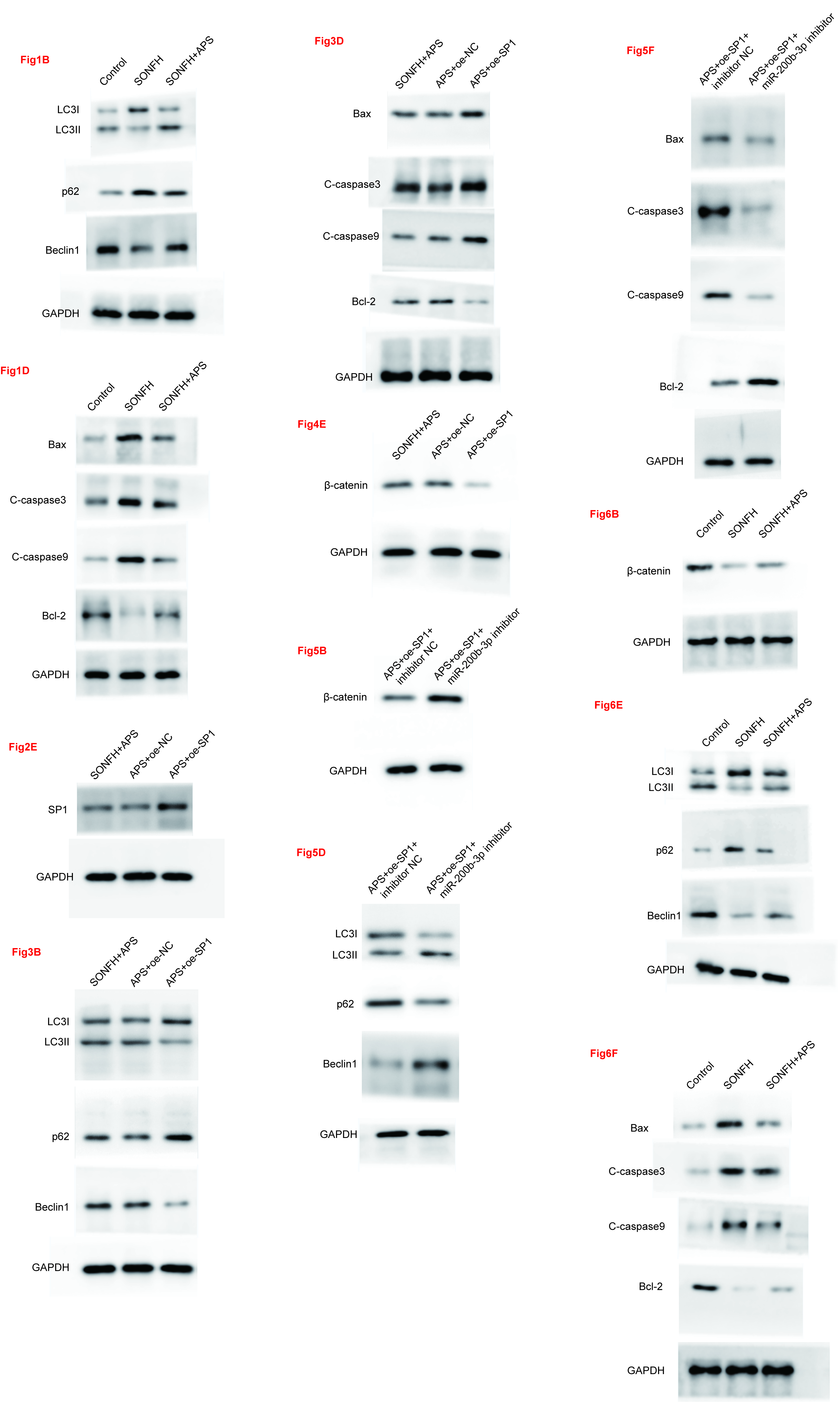

Supplement: Supplementary file 1 — Supplementary Material 1 [file 12891_2023_6447_MOESM1_ESM.tif]
